# Conductance artery stiffness impairs atrio-ventriculo-arterial coupling before manifestation of arterial hypertension or left ventricular hypertrophic remodelling

**DOI:** 10.1038/s41598-021-93614-w

**Published:** 2021-07-14

**Authors:** Kasper Kyhl, Sebastian von Huth, Annemie Bojer, Carsten Thomsen, Thomas Engstrøm, Niels Vejlstrup, Per Lav Madsen

**Affiliations:** 1 Department of Cardiology, Rigshospitalet, Copenhagen, Denmark; 2 Department of Cardiology, Herlev-Gentofte Hospital, Herlev, Denmark; 3 The MRI Section, Department of Radiology, Rigshospitalet, Copenhagen, Denmark; 4 Department of Clinical Medicine, University of Copenhagen, Copenhagen, Denmark

**Keywords:** Hypertension, Hypertension, Vascular diseases, Cardiac hypertrophy, Experimental models of disease

## Abstract

As part of normal ageing, conductance arteries lose their cushion function, left ventricle (LV) filling and also left atrial emptying are impaired. The relation between conductance artery stiffness and LV diastolic function is normally explained by arterial hypertension and LV hypertrophy as needed intermediaries. We examined whether age-related aortic stiffening may influence LV diastolic function in normal healthy subjects. Aortic distensibility and pulse wave velocity (PWV) were related to LV emptying and filling parameters and left atrial emptying parameters as determined by magnetic resonance imaging in 36 healthy young (< 35 years) and 16 healthy middle-aged and elderly (> 35 years) with normal arterial blood pressure and myocardial mass. In the overall cohort, total aorta PWV correlated to a decrease in LV peak-emptying volume (r = 0.43), LV peak-filling (r = 0.47), passive atrial emptying volume (r = 0.66), and an increase in active atrial emptying volume (r = 0.47) (all *p* < 0.001). PWV was correlated to passive atrial emptying volume even if only the > 35-year-old were considered (r = 0.53; *p* < 0.001). Total peripheral resistance demonstrated similar correlations as PWV, but in a regression analysis only the total aorta PWV was related to left atrial (LA) passive emptying volume. Via impaired ventriculo-arterial coupling, the increased aortic PWV seen with normal ageing hence affects atrio-ventricular coupling, before increased aortic PWV is associated with significantly increased arterial blood pressure or LV hypertrophic remodelling. Our findings reinforce the existence of atrio-ventriculo-arterial coupling and suggest aortic distensibility should be considered an early therapeutic target to avoid diastolic dysfunction of the LV.

## Introduction

Increased aortic pulse wave velocity (PWV;^1–3^) and increased left atrial (LA) volume are both statistically independent predictors of future cardiovascular disease^4^. In their milder forms, increased PWV as well as increased LA emptying problems are both part of normal ageing. Thus, with age conductance arteries loose distensibility^5^ and also the left ventricle (LV) develops early signs of diastolic dysfunction^6^. The impact on the heart of an increased PWV is explained by the pulse wave of stiff conductance arteries being reflected back from the periphery towards the heart, hence adding to LV myocardial stress during end-systole^7,8^. Indices of impaired ventriculo-arterial coupling is present in patients with LV hypertrophy and diastolic dysfunction in patients with heart failure with preserved ejection fraction (HFpEF)^9,10^. In this setting, LV diastolic dysfunction with impaired LA emptying and enlargement is seen and is explained by myocardial hypertrophic remodelling or ischemic heart disease as intermediaries via an eventual increase in blood pressure preceded by the stiffening of conductance arteries^10^. A correlation of high aortic PWV and signs of LV diastolic dysfunction in patients with hypertrophy have indeed been noted earlier^7,11–14^.

If aortic distensibility can be demonstrated to impact on LV diastolic mechanics without LV myocardial hypertrophy or signs of atherosclerosis as needed intermediaries to produce LV diastolic dysfunction, this will support the hypothesis that aortic distensibility is considered an important early therapeutic target for HFpEF^15^. With cardiovascular magnetic resonance imaging (CMR)^14^, aortic PWV and distensibility was determined in healthy young and middle-aged and elderly subjects with normal LV myocardial mass, without arterial hypertension or other known risk factors for atherosclerosis, in order to correlate PWV with early signs of LV diastolic dysfunction and impaired atrio-ventricular coupling. We aimed at an equal number of men and female gender as lower values for PWV are seen in women^16^ and controlled for variables associated with flow including hemoglobin^17,18^. We hypothesized that stiffening of the aorta with age is related to CMR indices of impaired LV filling and impaired LA function without the need for an increase in arterial pressure or LV hypertrophy to act as needed intermediaries (atrio-ventriculo-arterial coupling)^19^. If this were so this would add to thinking about a stiff aorta as an early therapeutic target to avoid later in life heart failure with preserved ejection fraction.

## Materials and methods

36 healthy young (< 35 years; 21 males) and 16 healthy middle-aged subjects (> 35 years; 8 males) were included. Exclusion criteria included pregnancy, a history of cardiovascular disease, hypertension (> 140/90 mmHg, or using anti-hypertensive medication), diabetes mellitus, coronary or peripheral artery disease, reduced kidney function, a history of systemic or connective tissue disease. Patients with known general inflammatory disease and immune-mediated disease were also excluded and disease states were screened for as all patients had normal C reactive protein. The study adhered to the principles of the Helsinki declaration. The study has been approved by the Regional Committees on Health Research Ethics in the Capital Region of Denmark (protocol: H-3-2012-074). All subjects gave written informed consent. Lipids (total cholesterol, low-density lipoprotein, high-density lipoprotein and triglycerides), HbA_1_c and hemoglobin concentration were measured in venous plasma, and left brachial artery systolic (SAP) and diastolic (DAP) blood pressures was measured in the resting supine state by a sphygmomanometer (Colin Press-Mate™ BP-8800; Colin Medical Instruments Corp., San Antonio, Texas, USA). Pulse pressure (PP) was calculated as the difference between SAP and DAP, and the mean arterial pressure (MAP) was determined as DAP + 1/3 of the PP.

### CMR protocol

CMR images were acquired on a 1.5-Tesla MRI scanner (Magnetom Espree, Siemens Medical Solutions, Erlangen, Germany) equipped with a 16-channel coil (retrospective ECG gating). Following scout imaging, steady-state free precession (SSFP) cine images were acquired corresponding to 2-, 3-, and 4-chamber followed by a short axis stack including atria as well as ventricles (slice thickness 8mm without gap; echo time 1.57ms; repetition time 50.24 ms; flip-angle 60°–70°; FOV-read 300–360 mm) with parallel imaging technique (GRAPPA, acceleration factor 2). Images were required during 8–10 s expiratory breath-holds with 25 phases of the cardiac cycle (temporal resolution of 24–50 ms). For aortic distensibility, two additional SSFP sequences were acquired in the transverse plane at the level of the pulmonary artery perpendicularly cross-cutting the ascending and descending aorta, and at the level of the abdominal aorta immediately below the diaphragm and above the renal arteries. If the abdominal aorta was not parallel to the long axis of the subject, the scanning plane was tilted so the abdominal aorta could be imaged perpendicularly. Through-plane flow sequences (free-breathing phase-contrast gradient-echo pulse sequences with velocity encoding, VENC 160m/s) were acquired in the ascending, descending, and abdominal aorta with the same scan planes as used for distensibility measurements. For determination of distance between scanned areas of the aorta, a sagittal SSFP-sequence image of the aorta was acquired covering its full course in the thorax and abdomen (‘candy cane’).

### Image analysis and calculations

CMR analysis was performed using dedicated software (CVi42 version 4.0.1, Circle Cardiovascular Imaging Inc., Calgary, Alberta, Canada). LV volumes were measured throughout the cardiac cycle from the short axis cine images using semi-automated edge detection, with the volume of each ventricular slice determined by drawing the endocardial contour (including papillary muscles in the myocardium) in all 25 phases. The volume of each slice was calculated as luminal area times slice thickness, and the sum of all slice volumes was used to obtain LV volumes. The LV stroke volume (SV) was the difference between the LV end-diastolic volume (EDV) and the LV end-systolic volume (ESV). LV ejection fraction (EF) was the LVSV divided by LVEDV. Cardiac output (CO) was calculated as the product of LVSV and heart rate (HR). LV peak-ejection (ER_p_) and peak-filling (FR_p_) rates for the LV were automatically calculated by the software as the maximal down- and early up-ward slopes of the LV time-volume curve and indexed to LVEDV. ER_p_ and FR_p_ were further indexed to BSA as ER_p(i)_ and FR_p(i)_. The fraction of the cardiac cycle duration for end-systole was determined to ascertain if end-systole occurred at the same time point in all, and LV stroke work (SW; g*m/m^2^) was determined as the LVSV indexed to body surface area multiplied by mean LV systolic pressure from the formula LVSW = LV mean systolic pressure * LVSV_(i)_ * 0.0136. The LV mean systolic pressure was determined as 0.9 * SAP^20^.

Arterial distensibility is the relative change in luminal cross-sectional area for a given change in PP and was calculated in the ascending, descending, and abdominal aortic lumen, respectively. With A_max_ and A_min_ denoting maximal and minimal aortic luminal areas (in mm^2^), distensibility is calculated as (A_max_-A_min_) * 100%/(A_min_ * PP) (in %/mmHg)^21^. Aortic PWV was calculated from the flow images by measuring the time delay, Δt, for the arterial waveform to pass a given length, Δx, between two aortic points, i.e. PWV = Δx/Δt^22^. The arrival of the aortic pulse wave was determined as the intersection of the tangent of the upslope of the flow curve to the time axis, as tangents were determined by the least-squares method of the three points surrounding the half-maximal flow. ∆t was determined as the difference between time-axis intersections of the ascending, descending, and abdominal aorta flow curves, respectively. PWV was calculated for the aortic arch, and for the total aorta. There is good agreement between PWV measured invasively and by CMR^23^.

LA maximal (LA_max_) and minimal volume (LA_min_) were determined from the short axis cine images. The LA mid-diastolic volume (LA_mdv_) and the LA volume immediately before atrial contraction (LA_lav_) were determined, and from these the LA passive emptying volume (LA_pev_) was determined as LA_max_-LA_mdv_. The LA_pev_ is a measure of the volume of blood flowing into the LV immediately after opening of the mitral valve as a consequence of LV early diastolic filling. The LA active emptying volume (LA_aev_) was LA_lav_-LA_min_, as a measure of the volume of blood flowing into the LV during late ventricular diastole as a consequence of the active systole of the LA (“atrial kick”). LA conduit volume (LA_cv_), a measure of the blood that flows through the LA passively during LV diastasis, was calculated as LVSV-(LA_pev_ + LA_aev_). LA reservoir volume (LA_rv_) was calculated as LA_pev_ + LA_aev_. LA passive emptying fraction (LA_pef_) was calculated as LA_pev_/LA_max_. LA active emptying fraction (LA_aef_) was calculated as LA_aev_/LA_lav_. LA reservoir emptying fraction (LA_ref_) was calculated as (LA_max_-LA_min_)/LA_max_.

### Statistical analyses

Normally distributed data are expressed as mean ± SD, and non-normally distributed data are expressed as median with range. LV and LA volumes were indexed to body surface area (Mosteller)^24^. Total peripheral resistance (TPR) was calculated as MAP divided by CO. Data were tested visually for normal distribution. Normal distributed data were analyzed using ANOVA, and if significant the Tukey post-hoc test was used to identify which group means differed. Correlations were evaluated with Pearson’s r. Independent influence of variables on FR_p(i) and_ LA_PEV_ was evaluated with least square multiple variable regression analysis. Age, sex, and MAP were considered as independent variables, however, age and sex highly influenced both TPR and PWV_Total_ and MAP was highly correlated with TPR and were therefore excluded as independent variables. Statistical significances were indicated by a *p* ≤ 0.05. Statistical analysis was performed using the SPSS software package (IBM SPSS v19.0 for Mac, SPSS Inc., Chicago, IL, USA).

## Results

Male subjects had a higher BSA than females, and BSA increased with age, but besides BSA no significant differences were noted between genders and age groups for HR, SAP, DAP, PP or any of the blood chemistry parameters (Table 1). All baseline characteristics were within the normal range for age and gender.

**Table 1:** Baseline characteristics for 36 healthy young and 16 healthy middle-aged subjects studied with CMR to determine the relationship of aortic distensibility to left ventricular diastolic function.

	Men < 35 years	Women < 35 years	Men > 35 years	Women > 35 years
N	21	15	8	8
Age, years	26 (20–32)	26 (19–33)	58 (50–65)**	59 (51–66)**
BMI, kg/m^2^	23.4 (2.7)	22.3 (3.4)	27.1 (2.6)*	24.0 (1.7)
BSA, m^2^	2.0 (0.2)	1.7 (0.1)^##^	2.2 (0.2)*	1.8 (0.1)^##^
Heart rate, bpm	63 (11)	71 (10)	57 (7)	67 (10)
SAP, mmHg	123 (12)	127 (11)	130 (9)	129 (21)
DAP, mmHg	65 (8)	69 (8)	72 (8)	71 (9)
PP, mmHg	58 (7)	57 (8)	57 (5)	59 (13)
TPR, mmHg min/L	11.1 (1.7)	13.6 (3.4)	13.5 (1.6)	15.8 (1.9)
Chol._Total_, mmol/L	4.6 (0.6)	4.6 (1.0)	5.3 (0.9)	5.4 (0.6)
HDL, mmol/L	1.6 (0.4)	1.8 (0.4)	1.5 (0.3)	2.2 (0.4)^##^
LDL, mmol /L	2.7 (0.6)	2.5 (0.8)	3.3 (1.0)	3.1 (0.8)
Triglyceride, mmol/L	1.37 (0.71)	1.22 (0.60)	1.71 (0.71)	0.97 (0.38)
Hemoglobin, mmol/L	9.3 (0.5)	8.4 (0.4)^##^	9.3 (0.3)	8.5 (0.5)^##^
Hct, %	43 (2)	40 (1)^##^	44 (2)	41 (3)
Albumin, g/L	49 (3)	45 (2)^##^	45 (2)*	45 (2)
CRP, mg/L	1.1 (0.4)	3.3 (3.2)^#^	1.5 (1.8)	1.0 (0.0)*
Glucose, mmol/L	5.7 (0.4)	5.8 (0.3)	6.0 (0.4)	6.3 (0.5)*

*p < 0.05 and **p < 0.01 vs. young of same gender; ^#^*p* < 0.05 and ^##^*p* < 0.01 vs. different gender of similar age.

**Figure 1:**
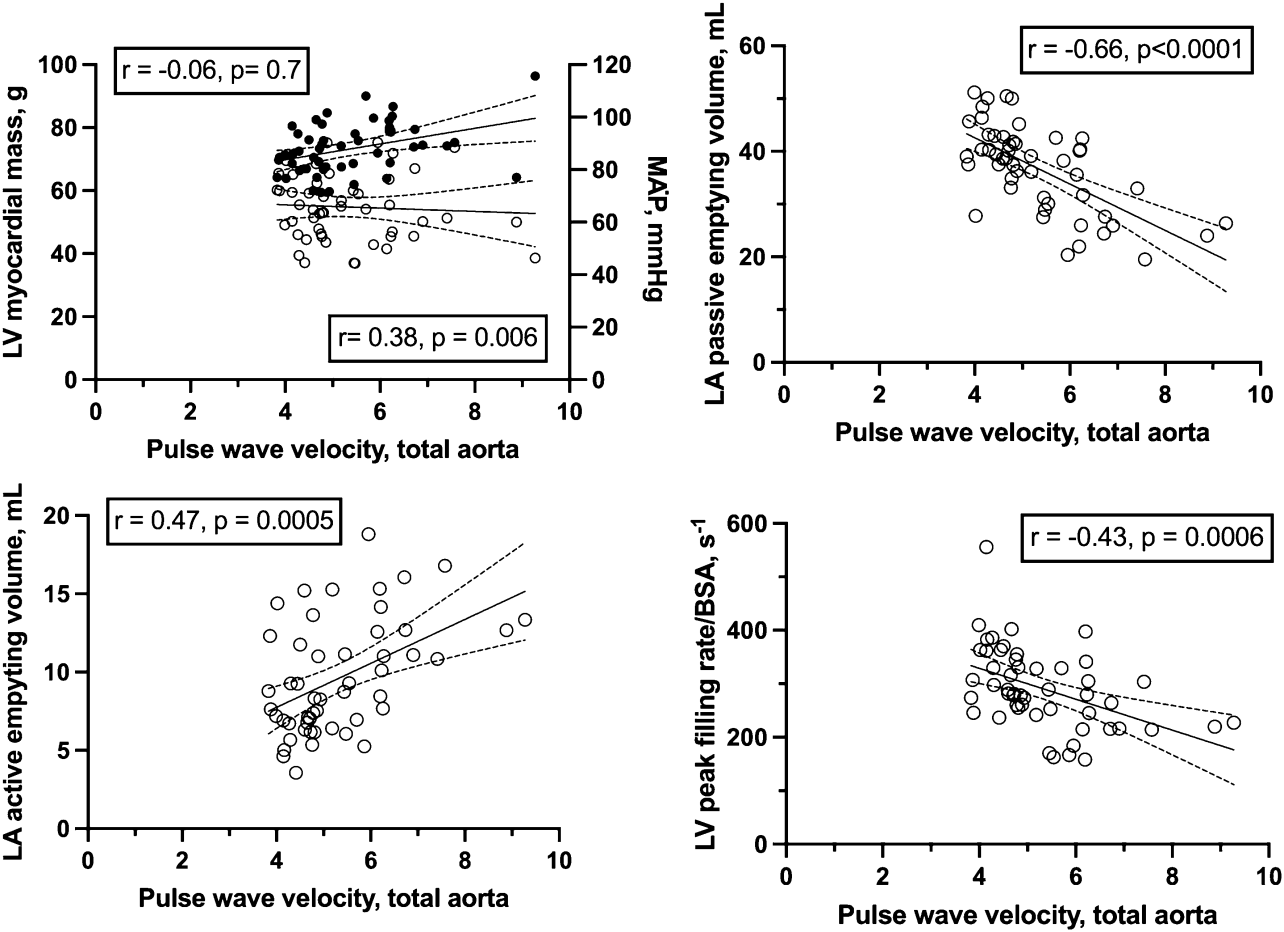
Upper left, left ventricular (LV) myocardial mass (circles, left axis) and mean arterial pressure (MAP; solid circles, right axis) to total aortic pulse wave velocity (circle) as determined from CMR time-volume curves in 36 healthy young and 16 healthy middle-aged subjects. Lower left, left atrium (LA) active emptying volume to total aortic pulse wave velocity. Upper right, LA passive emptying volume to total aortic pulse wave velocity. Lower right, indexed LV peak filling rate to total aortic pulse wave velocity. Total aortic pulse wave velocity is correlated to LV peak filling rate (r = − 0.47) and both LA passive (r = − 0.66) and active emptying volumes (r = 0.47) (all *p* < 0.05).

All volumetric characteristics of the LV and LA were within normal reference intervals and show slightly lower LVEDV in women (Table 2). There was no age- or gender-related differences in LVEF, and myocardial mass was lower in women, but not related to age, whereas both ER_p_ and FR_p_ decreased with age in both genders (Table 2), and LA_max_, LA_min_, LA_PEV_ and LA_PEF_ decreased with age (Table 2). Without significant gender differences, aortic distensibility of the ascending, descending and abdominal aorta decreased with age, and distensibility was highest in the abdominal aorta (Table 2). Inversely related to aortic distensibility (Fig. 1), PWV increased with age both in the aortic arch and in the total aorta (Table 2).

**Table 2:** Left ventricle and left atrial variables as determined from CMR time-volume curves in 36 healthy young and 16 healthy middle-aged subjects.

	Men < 35 years	Women < 35 years	Men > 35 years	Women > 35 years
LVEF, %	57 (4)	61 (4)	58 (4)	63 (4)*
Frac._End-systole time_	0.36 (0.08)	0.36 (0.08)	0.36 (0.08)	0.36 (0.08)
LVEDV_(i)_, mL/m^2^	101 (13)	84 (8)^##^	91 (9)	75 (9)^##^
LVESV_(i)_, mL m^−2^	43 (8)	33 (4)^##^	38 (6)	28 (5)^##^
LVSV_(i)_, mL/m^2^	58 (7)	51 (8)^#^	52 (5)	47 (5)
CI, L/min/m^2^	3.9 (0.7)	3.9 (1.1)	3.2 (0.5)	3.3 (0.6)
LVSW, g*m/m^2^	87 (13)	79 (15)	83 (8)	75 (15)
Myo. mass_(i)_, g/m^2^	61 (8)	47 (6)^##^	64 (10)	43 (5)^##^
LVmass/EDV	0.6	0.55	0.7	0.57
LV ER_p_/EDV	3.1	3.4	2.8	3
ER_p(i)_, mL min^−1^ m^−2^	315 (37)	283 (60)	259 (27)**	227 (30)**
FR_p_/LVEDV	3.03	4.1	2.3	3.2
FR_p(i)_, mL/min^/^m^2^	307 (45)	344 (88)	208 (38)**	241 (42)**
FR_p_/FR_p_	0.97	1.21	0.8	1.06
LA_max(i)_, mL m^−2^	46 (9)	42 (6)	53 (15)	47 (8)
LA_min(i)_, mL m^−2^	22 (5)	19 (4)	29 (10)^##^	22 (5)
LASV_(i)_, mL m^−2^	24 (5)	24 (4)	24 (5)	25 (5)
LAEF, %	53 (6)	56 (7)	46 (6)^#^	53 (6)
LA_mdv(i)_, mL m^−2^	28 (7)	24 (5)	40 (13)**	33 (6)**
LA_LDV(i)_, mL m^−2^	31 (8)	26 (5)	42 (14)**	34 (6)**
LA_pev(i)_, mL m^−2^	18 (4)	18 (3)	13 (2)**	14 (3)**
LA_aev(i)_, mL m^−2^	9 (3)	7 (2)	13 (3)**	12 (3)**
LA_cv(i)_, mL m^−2^	30 (5)	26 (5)	26 (4)	21 (5)
LA_rv(i)_, mL m^−2^	27 (6)	25 (4)	26 (5)	26 (5)
LA_pef_, %	40 (6)	43 (5)	25 (4)**	30 (4)**
LA_aef_, %	29 (6)	28 (5)	32 (4)	34 (7)
Aorta_Asc_., %	33 (9)	44 (14)^#^	15 (7)**	13 (8)**
Aorta_Desc_., %	30 (7)	31 (5)	20 (6)**	19 (5)**
Aorta_Abd_., %	45 (10)	47 (10)	27 (5)**	23 (6)**
D_Aorta Asc_	5.8 (1.5)	7.9 (2.9)^#^	2.5 (1.1)**	2.2 (1.3)**
D_Aorta Desc_	5.2 (1.1)	5.6 (1.3)	3.4 (0.9)**	3.4 (1.2)**
D_Aorta Abd_	7.8 (1.7)	8.3 (2.2)	4.7 (0.8)**	4.0 (1.5)**
PWV_Arch_, m/s	6.3 (1.9)	5.4 (1.3)	8.9 (3.7)	8.4 (3.2)*
PWV_Total_, m/s	4.7 (0.6)	4.8 (0.8)	6.8 (1.1)**	6.4 (1.4)**

*p < 0.05 and **p < 0.01 vs. young of same gender; ^#^*p* < 0.05 and ^##^*p* < 0.01 vs. different gender of similar age.

### Correlation and multiple regression analysis

TPR correlated with ER_p(i)_, FR_p (i)_, and LA_PEV_, and myocardial mass correlated with ER_p(i)_ and LA_max_ (Table 3). Distensibility of the aorta and PWV of the aortic arch and the total aorta correlated with each other (data not shown), and with FR_p_, LA_PEF_ and LA_AEF_, but not with LV myocardial mass, ER_p_ or LA_max_ (Table 3, Fig. 1). LA_PEV_ and PWV_Total_ were inversely correlated (r = − 0.66 og *p* < 0.0001) even if only the > 35 years were included.

**Table 3:** Correlations of arterial characteristics to variables related to LV systolic and diastolic function.

	Myo. mass	ERp(i)	FRp(i)	LAMAX	LAPEV	LAAEV
MAP	0.04	0.03	− 0.04	− 0.01	− 0.11	− 0.01
TPR	− 0.25	− 0.52**	− 0.44**	0.03	− 0.31*	0.22
Myocardial mass	–	0.34*	0.08	0.55**	− 0.007	0.37**
DAorta Ascendens	0.02	0.32*	0.45**	− 0.17	0.57**	− 0.47
DAorta Descendens	0.05	0.13	0.26 (*p* = 0.06)	− 0.22	0.53**	− 0.51**
DAorta Abdominal	− 0.03	0.15	0.39**	− 0.16	0.47**	− 0.45**
PWV Arch	− 0.15	− 0.25	− 0.33*	− 0.04	− 0.34	0.17
PWV Total	− 0.06	− 0.43**	− 0.43**	0.18	− 0.66**	0.47**
DAorta Ascendens, < 35 years	− 0.17	− 0.04	0.08	0.04	0.18	− 0.21
PWV Total, > 35 years	0.02	0.04	0.02	0.02	− 0.53**	0.15

Correlations of blood pressure (MAP), total peripheral resistance (TPR), Pulse wave velocity (PWV) and aortic distensibility (DAorta) variables to variables related to LV systolic (ERp(i)) and diastolic (FRp(i)) function and left atrial maximal volume (LAMAX) and passive (LAPEV) and active (LAPEV) emptying parameters and *p < 0.05 and **p < 0.01.

For the least square multiple variable regression analysis, TPR and PWV_Total_, both independently affected PFR/BSA (Table 4) whereas only PWV_Total_ independently affected the LA_PEV_ (Table 5). In Goodness of Fit analysis, R^2^ values were 0.3105 for the model of PFR/BSA and 0.4221 LA_PEV_, respectively.

**Table 4:** Least square multiple regression analysis for left ventricle peak-filling rate indexed (FR_P(i)_) as determined from CMR time-volume curves in 36 healthy young and 16 healthy middle-aged subjects (unstandardized coefficients).

	B	Std. Error	t	Significance level	Collinearity statistic VIP
Constant	528.7	54.65	9.674	< 0.001	
TPR	− 9.811	3.939	2.491	< 0.05	1.45
PWVTotal	− 20.67	8.447	2.447	< 0.05	1.45

**Table 5:** Least square multiple regression analysis for left atrial passive emptying volume (LA_PEV_) as determined from CMR time-volume curves in 36 healthy young and 16 healthy middle-aged subjects (unstandardized coefficients).

	B	Std. Error	t	Significance level	Collinearity statistics VIP
Constant	62.97	5.464	11.53	< 0.0001	
TPR	− 0.3471	0.3939	0.8813	ns	1.45
PWVTotal	− 4.052	0.8445	4.798	< 0.0001	1.45

## Discussion

We investigated to what degree a change in aorta distensibility affects atrio-ventriculo coupling in normal subjects without arterial hypertension or LV hypertrophic remodelling. Aortic distensibility and PWV were related to LV filling patterns and early signs of LA emptying impairment with CMR in a cohort of normal young and middle-aged subjects. Thus, age-related decrease in aortic distensibility and increase in PWV are correlated to a decrease in not only LV peak-filling but also passive LA emptying volume with a correspondent increase in active atrial emptying volume. In multiple variable regression analysis both total peripheral resistance and PWV_Total_ independently influenced LV peak-filling rate. PWV_Total_ independently accounted for 42% of LA_PEV_ changes. PWV and LA passive emptying volume correlated even if only the > 35-year-old were considered. CMR measured LV filling and LA volume and emptying parameters are well established as indicators of diastolic dysfunction and our results point to aortic stiffness being of independent importance for LV filling impairment^19,25^. The clinical importance of this relationship should be confirmed by longitudinal data.

Conductance arteries ideally transform the pulsatile LV outflow into a smooth continuous flow in the peripheral capillaries, as during systole aorta distends and thereby stores energy generated (“Windkessel”). The pulse wave, however, travels forward quickly, and theory suggests the pulse wave is reflected back from the periphery towards the heart against the forward moving blood. With distensible conductance arteries, PWV is low, and pulse wave reflection therefore will be small and will arrive after the aortic valve has closed hence not affecting LV. With stiffer conductance arteries, PWV increases, and the reflected wave returns before the aortic valve has closed and thus help increase late-systolic arterial blood pressure and afterload for the LV. Thus, whereas the TPR reflects hydraulic opposition to LV outflow during early LV systole, the reflected pulse wave becomes an added burden to the LV during late systole. In normal subjects, aortic distensibility decreases already in the 3th–4th decade, followed by an increase in aortic PWV and the DAP and PP beyond the 6th decade^5,26^. Even without increased DAP and PP, however, normal cohort studies with CMR demonstrate stiffer LV as determined by decreased FR_p_, and impaired LA_PEV_, and our results now show that quantitative measures of conductance artery stiffness and high PWV correlate with indicators of diastolic dysfunction. Such changes, independently of any LV hypertrophy or increments in blood pressure or TPR (i.e. besides the late-systolic increase seen with the reflected PW), can affect LV function as has recently been suggested for blood pressure^27^, and has been shown for athletes using anabolic steroids^28^. Increased afterload, impacted by stiff conductance arteries, early impacts entire atrio-ventricular-arterial coupling cascade, and does not need to wait years for an increase in PP or LV hypertrophy to become manifest. In cohort studies of patients with HFpEF, high PWV is already noted to be prevalent, but these patients also have increased PP and LV hypertrophy to account for LV diastolic dysfunction and signs of heart failure^7,11–14^, and therefore from these studies it is less obvious that PWV is of independent direct importance for the atrio-ventriculo-arterial coupling cascade. In different disease models, a negative correlation between PWV and left atrial strain has been shown^29,30^. With all important variables measured on precise quantitative scales, our study now establish that now only is PWV in subjects were FR_p_, LA_pev_ and LA_aev_ are affected, but PWV is in fact correlated with not only peak LV ejection rate but also LV relaxation with consequent LA emptying changes well before LV hypertrophy or PP increments. Hence age-related changes on impaired LV relaxation with lowered FR_p_ and impaired LA_PEV_ reported in normal materials of young and elderly subjects is partly explained by stiffening of the conductance arteries. With a stiff aorta demonstrably impacting on the entire atrio-ventriculo-arterial coupling sequence even in normal young and elderly. We cannot say by what mechanism increased aortic stiffness impinges on the LV filling rate. We speculate that part of the mechanism is by LV myocardial ATP-depletion (and if so it would be immediately correctable), part by induction of LV myocardial diffuse fibroses (and if so it would be more important to avoid in good time)^31^.

### Strengths and limitations

It is a strength of our study that we only invited healthy subjects without hypertension or LV myocardial hypertrophy. Also, it is a strength that we applied CMR, the reference standard for non-invasive flow and cardiac volumes, to determine parameters of importance on the same study day. CMR also provides excellent reproducibility of peak filling and peak emptying rate^32,33^. It is a limitation that our study is cross-sectional. In theory, it may therefore be that aortic stiffness, ER_p(i)_ and FR_p(i)_ and LA_PEV_ co-vary not because of causality, but because they were affected by the same overarching variable. Atherosclerosis would be a candidate for such an overarching variable. However, no subject had signs of or risk factors for ischemic heart disease, and such would have to be significant to lower FR_p_ that in our study was comparable to FR_p_ recorded in other normal materials. Further studies are warranted to investigate the relation between diastolic function and diffuse myocardial fibrosis by T1 mapping in healthy as this was not performed in the present study. To the best of our knowledge healthy subjects – in opposition to for example patients with HFPEF, hypertension or diabetes – are not known to have significant myocardial fibrosis^31,34,35^.

## Conclusion

Increased aortic stiffening and pulse wave velocity impairs atrio-ventriculo-arterial coupling before manifest arterial hypertension or left ventricular hypertrophic remodelling is seen. Our findings provide physiological rational for designing randomized studies to target aortic distensibility as an early therapeutic target to avoid later in life HFpEF.

## Abbreviations

CMR: Cardiovascular magnetic resonance imaging
ER_p_: Peak-ejection rate
FR_p_: Peak-filling rate
LA: Left atrium
LA_aef_: Left atrium active emptying fraction
LA_aev_: Left atrium active emptying volume
LA_lav_: Left atrium volume before atrial contraction
LA_cv_: Left atrium conduit volume
LA_max_: Left atrium maximal volume
LA_mdv_: Left atrium mid-diastolic volume
LA_min_: Left atrium minimal volume
LA_pef_: Left atrium passive emptying fraction
LA_pev_: Left atrium passive emptying volume
LA_ref_: LA reservoir emptying fraction
LA_rv_: Left atrium reservoir volume
PWV: Pulse wave velocity
